# Supplementary material for: Dynamic interactions of influenza viruses in Hong Kong during 1998-2018
Source: PLoS Comput Biol. 2020 Jun 15;16(6):e1007989. doi: 10.1371/journal.pcbi.1007989 (PMC7316359; doi:10.1371/journal.pcbi.1007989)
Supplement: S1 Text — (DOCX) [file pcbi.1007989.s001.docx]

**S1 Text. Supporting Information**

**Dynamic interactions of influenza viruses in Hong Kong during 1998-2018**

Wan Yang, Eric H. Y. Lau, Benjamin J. Cowling

This supplemental document includes 1) Validation of the model-inference system using model-generated mock epidemics; 2) Simulations to test the impact of cross-immunity on long-term epidemic pattern; and supplemental table and figures; and 3) algorithm for the multi-strain SIRS model-SR-IF2 inference system.

**1. Validation of the model-inference system**

1.1 Method

We first tested our multi-strain SIRS model-SR-IF2 inference system using model-generated mock epidemics (i.e. synthetic data). Specifically, we tested its ability to estimate three main epidemic features of interest: 1) The population susceptibility for each virus over time, including timing of major susceptibility increase due to epochal antigenic change. For the latter, we tested three levels of magnitude—small, medium, and large change—with 10%, 15%, and 25% increase in susceptibility, respectively. 2) The strength of cross-immunity between each virus-pair. For this, we tested two combinations of heterosubtypic immunity (i.e., low H3N2→H1N1 and high H1N1→H3N2 cross-immunity for the first synthetic dataset; high H3N2→H1N1 and median H1N1→H3N2 cross-immunity for the second). and 3) The duration of immunity. Reported immunity period for influenza varied substantially, ranging from months to ~8 years (1-4). Thus, we tested prior ranges from 1 to 9 years.

To generate the mock epidemics for the above testing, we first generated 100,000 random combinations of initial conditions and model parameters except *R_0_*, using Latin Hypercube sampling. Weekly *R_0_* values were randomly sampled from a normal distribution with the mean set to the empirical estimate for that week (Fig 1 in the main text) and standard deviation set to 5% of the mean. To mimic punctuated antigenic changes, for each (sub)type, we first identified the observed major epidemic peak weeks (peak ILI+ in the upper ~75%, 60%, and 75% percentiles for the three (sub)types, respectively); for each selected peak week, we increased the susceptibility at the 8^th^ preceding week (i.e., roughly the onset) by 15% for A(H1N1) (except for the pandemic) and A(H3N2), by 10% for B, and by 25% for the A(H1N1) pandemic (see the selected weeks in S2-3 Figs). We then ran the multi-strain SIRS model (Eqn 1 in the main text) for each setting and selected 2 simulations that most closely resembled the observed epidemics in Hong Kong but had different levels of cross-immunity between the two A subtypes: the first simulated time series had high cross-immunity from A(H1N1) against A(H3N2) but low cross-immunity in the reverse direction; and the second had moderate cross-immunity from A(H1N1) against A(H3N2) and high cross-immunity from A(H3N2) against A(H1N1). We then added Poisson random noise to the two simulated time series to mimic observational errors and used these as mock data (termed synthetic 'truth') to test the model-inference system.

For each of the two synthetic truths, we performed two rounds of model-inference using the same procedure and prior distributions for the Hong Kong dataset as described in the main text. To evaluate the accuracy of the final posterior estimates, we computed the root-mean-square-error and correlation with the 'truth'.

*1.2 Results*

S2-3 Figs show the posterior estimates of key model state variables (population susceptibility for the three types/subtypes) and parameters (the basic reproductive number *R_0_*, infectious period, immunity period and strength of cross-immunity) for the two tests, respectively, compared with the true values. The relative root-mean-square-error was 0.16 and the correlation was 0.83 combining all model variables/parameters from both tests. These results suggest that our model-inference system and parameter estimation strategy is able to accurately estimate the model state variables and parameters. However, due to large fluctuations of *R_0_* in the synthetic datasets (e.g., S2 Fig, B) and the collinearity between *R_0_* and susceptibility, the filter tended to compensate sudden increases in infections (due to increases in susceptibility to mimic punctuated antigenic changes) with increases in *R_0_* rather than susceptibility. As a result, it failed to identify some of the punctuated antigenic changes, in particular, for the two A subtypes with larger variances of *R_0_*.

**2. Simulating the impact of cross-immunity on long-term epidemic pattern**

*2.1 Method*

To test the impact of cross-immunity, we simulated epidemics under three different scenarios: 1) As estimated in this study, by setting all cross-immunity terms to the posterior mean estimates; 2) Stronger cross-immunity from A(H3N2) against A(H1N1), by setting *c_H1←H3_* to 0.5, i.e., slightly above the mean estimate for *c_H3←H1_* (i.e. 0.4); and 3) No interactions, by setting all cross-immunity terms to 0. For each simulation, we initiated the multi-strain SIRS model (Eqn 1 in the main text) using the posterior estimates at Week 4 (i.e., after discarding the first three weeks of filter spin-off) and ran the model stochastically from Jan 1998 to July 2018. Weekly values of model parameters (i.e., the infectious period, immunity period, *R_0_*, and cross-immunity terms) not specified in the above scenarios were set to the mean posterior estimates from the Hong Kong dataset. Weekly susceptibilities to the three types/subtypes were simulated by the model according to model-simulated infections, loss of immunity, cross-immunity, replenishment from newborns, and deaths; however, to simulate the impact from antigenic innovations, we reset the susceptibility to the posterior mean estimate from the Hong Kong dataset for weeks identified to experience punctuated antigenic changes (Table 1). To account for model stochasticity, we simulated each scenario for 1000 times.

To compute the number of epidemics over the study period, as in (5), we defined the epidemic baseline, for each type/subtype, as the 40% quantile of the non-zero corresponding ILI+ records over the ~20 year study period. We then identified 1) all epidemics including small ones, defined as periods with ≥3 consecutive weeks with ILI+ above the baseline and at least one of the weeks with ILI+ ≥3 times of the baseline; and 2) all large epidemics, defined as periods with ≥6 consecutive weeks with ILI+ above the baseline and at least one of the weeks with ILI+ ≥6 times of the baseline. If an identified epidemic lasted for >1 year, we divided it into multiple epidemics at the year division(s). Based on the 1^st^ definition, there were 17 A(H1N1), 21 A(H3N2), and 18 B epidemics, respectively (S4 Fig); and based on the 2^nd^ definition, there were 13, 19, and 14 epidemics of the three types/subtypes, respectively, during Jan 1998—July 2018 (S5 Fig). We used the same baselines as observed and definitions to identify and compute the numbers of epidemics in the simulations and compared them to the observed.

To compare the co-circulation patterns, we categorized the weeks into 8 possible co-circulation types: 1) none in circulation, i.e., none of the three types/subtypes had ILI+ above their baselines; 2) A(H1N1) alone, i.e., only A(H1N1) had ILI+ above its baseline; and similarly, 3) A(H3N2) alone and 4) B alone; 5) A(H1N1)+A(H3N2), i.e., only A(H1N1) and A(H3N2) had ILI+ above their baselines; 6) A(H1N1)+B, i.e., only A(H1N1) and B had ILI+ above their baselines; 7) A(H3N2)+B, i.e., only A(H3N2) and B had ILI+ above their baselines; and 8) All, i.e., all three types/subtypes had ILI+ above their baselines. We then computed the percentages of weeks falling in each of the 8 co-circulation types and compared them to the observed.

*2.2 Results*

Figures 8-10 show the model-simulated epidemic trajectories compared to the observations in Hong Kong. As expected, without the constraints by observations (as in a model-inference system), the errors grew quickly and thus the simulated epidemics deviated from the observations. However, overall, simulations with cross-immunity in place were able to better reproduce the observed epidemic pattern (S8-9 Figs vs. S10 Fig). In particular, those simulations were able to reproduce the long periods with near-zero A(H1N1) activities (e.g., 2002-2005) whereas simulations with no cross-immunity (S10 Fig) overestimated the circulation of all types/subtypes, especially for A(H1N1). Comparing the first two scenarios (i.e. both with cross-immunity), simulations with stronger cross-immunity from A(H3N2) against A(H1N1) underestimated the circulation of A(H1N1) and were only able to generate A(H1N1) epidemics following the simulated punctuated antigenic changes when the susceptibility was reset (S9 Fig vs. S8 Fig as estimated from the data).

Tally over the entire study period, the first two scenarios (i.e., with cross-immunity) appeared to underestimate the total number of the epidemics (S11 Fig A and B). However, this was due to the challenge in generating clear bimodal epidemics within a year as those observed in Hong Kong. For instance, there were two consecutive A(H1N1) epidemics in 2008; and similarly, A(H3N2) tended to cause two separate epidemics within a year, e.g., 1999, 2000, 2003, and 2014. The underestimation of number of A(H1N1) epidemics was severer for scenario 2 when stronger cross-immunity from A(H3N2) against A(H1N1) was implemented in the model. Simulations with no cross-immunity were able to generate more epidemics due to overestimation of circulation during periods without observed influenza activity (S10 Fig).

Comparing the 8 co-circulation types (Fig. 11C), scenario 1 using the posterior mean estimates most closely reproduced the observed pattern. In contrast, scenario 2 (i.e., stronger cross-immunity from A(H3N2) against A(H1N1)) largely underestimated the frequency of A(H1N1) epidemics, whereas scenario 3 (i.e., no cross-immunity) largely overestimated co-epidemics caused by multiple types/subtypes.

Further, we compared model simulations (run through the entire study period using the posterior parameter estimates) stratified by period before and after the 2009 A(H1N1) pandemic. As shown in S12 Fig, the simulated co-circulation patterns among the three influenza viruses similarly matched with the observed co-circulation patterns before and after the 2009 pandemic. This result suggests that the parameter posterior estimates were able to capture cross-immunity features before and after the 2009 A(H1N1) pandemic.

References:

1. Ferguson NM, Galvani AP, & Bush RM (2003) Ecological and immunological determinants of influenza evolution. *Nature* 422(6930):428-433.

2. Yaari R, Katriel G, Huppert A, Axelsen JB, & Stone L (2013) Modelling seasonal influenza: the role of weather and punctuated antigenic drift. *J R Soc Interface* 10(84):20130298.

3. Ranjeva S*, et al.* (2019) Age-specific differences in the dynamics of protective immunity to influenza. *Nat Commun* 10(1):1660.

4. Kucharski AJ*, et al.* (2015) Estimating the life course of influenza A(H3N2) antibody responses from cross-sectional data. *Plos Biol* 13(3):e1002082.

5. Yang W, Cowling BJ, Lau EH, & Shaman J (2015) Forecasting Influenza Epidemics in Hong Kong. *PLoS Comput Biol* 11(7):e1004383.
