# Supplementary material for: Dynamic interactions of influenza viruses in Hong Kong during 1998-2018
Source: PLoS Comput Biol. 2020 Jun 15;16(6):e1007989. doi: 10.1371/journal.pcbi.1007989 (PMC7316359; doi:10.1371/journal.pcbi.1007989)
Supplement: S1 Table — (DOCX) [file pcbi.1007989.s002.docx]

**S1Table.** Estimated timing of punctuated antigenic changes using a cutoff of 10% increase in susceptibility, instead of 15% in Table 2.

| Strain | Date | SD (days) |
| --- | --- | --- |
| A(H1N1) | 1/21/00 | 4.05 |
|  | 6/11/06 | 0.00 |
|  | 1/25/09 | 0.00 |
|  | 7/6/09 | 3.50 |
|  | 9/13/09 | 2.21 |
|  | 1/4/10 | 6.30 |
|  | 1/14/11 | 3.13 |
| A(H3N2) | 1/18/98 | 0.00 |
|  | 5/15/98 | 6.19 |
|  | 1/10/99 | 4.31 |
|  | 6/13/99 | 12.12 |
|  | 1/9/00 | 1.94 |
|  | 6/27/00 | 5.72 |
|  | 1/13/03 | 9.46 |
|  | 7/11/04 | 0.00 |
|  | 2/25/05 | 14.53 |
|  | 6/20/07 | 3.74 |
|  | 7/28/10 | 3.83 |
|  | 5/10/12 | 8.08 |
|  | 1/2/15 | 4.21 |
|  | 6/6/15 | 8.08 |
| B | 2/6/00 | 0.00 |
|  | 1/28/01 | 9.90 |
|  | 1/27/02 | 0.00 |
|  | 1/6/03 | 7.06 |
|  | 1/13/18 | 0.00 |
