# Supplementary material for: Dynamic interactions of influenza viruses in Hong Kong during 1998-2018
Source: PLoS Comput Biol. 2020 Jun 15;16(6):e1007989. doi: 10.1371/journal.pcbi.1007989 (PMC7316359; doi:10.1371/journal.pcbi.1007989)
Supplement: S2 Table — (DOCX) [file pcbi.1007989.s003.docx]

**S2 Table.** Main model parameters and prior ranges tested.

| Parameter | Symbol | Prior range | Source/rationale |
| --- | --- | --- | --- |
| Number of susceptible individuals for virus-*i* | *S_i_* | [50%, 90%] of total population for initial S | The study started from Week 1 of the year, at the beginning of an epidemic, so susceptibility was likely high |
| Number of infectious individuals for virus-*i* | *I_i_* | N/A | Estimated based on data |
| Number of recovered individuals for virus-*i* | *R_i_* (S13 Fig) | N/A | *R_i_* = *N* - *S_i_ - I_i_,* for *i*=H1, H3, or B |
| Population size | *N* | Set to 100,000 | Scaled, as the model outputs are rates per 100,000 population. |
| Basic reproductive number at week *t* | *R_0,i_*(*t*) | Estimated from the seasonal cycle | See main text. |
| Infectious period | *D_i_* | [2, 4] days |  |
| Transmission rate at week *t* | *β_i_*(*t*) | N/A | *β_i_* (*t*) *=R_0,i_*(*t*)/*D_i_*(*t*) |
| Immunity period | *L_i_* | [1, 9] years | Reported values range from months to ~8 years [76, 77]. |
| Strength of cross-immunity to virus-*i* conferred by infection of virus-*j* | *c_ij_* | [0%, 80%] of the specific immunity | Possible range [0, <100%] |
| Travel related importation (i.e. seeding) | *α* | Set to 1 per 10 days | Arbitrary low number |
| Birth/death rate | *μ* | Set to 1/75/365 | Assume a lifespan of 75 years |
