# Supplementary material for: Dynamic interactions of influenza viruses in Hong Kong during 1998-2018
Source: PLoS Comput Biol. 2020 Jun 15;16(6):e1007989. doi: 10.1371/journal.pcbi.1007989 (PMC7316359; doi:10.1371/journal.pcbi.1007989)
Supplement: S3 Table — (DOCX) [file pcbi.1007989.s004.docx]

**S3 Table.** IF2 algorithm with space reprobing.

| INITIATION: initiate the whole model system ***x***, including all parameters $\Theta_{0}$, by Latin Hypercube Sampling (LHS) from the prior bounds.  FOR m=1:M (iterations)  IF m=1, set $\Theta_{m}$=$\Theta_{0}$  ELSE add perturbation to the parameter distribution $\Theta_{m}\sim h_{m}(\theta\vert\Theta_{m-1};\delta_{m})$  END IF  1. Initialization for the particle filter: at k=0, ${\{\boldsymbol{x}_{0}^{i},w_{0}^{i}\}}_{i=1}^{N}$  $where, w_{0}^{i}=1/N$  2. FOR k=1:K (time steps)  - FOR i=1:N (particles)   - Draw $\boldsymbol{x}_{k}^{i}\sim\pi(\boldsymbol{x}_{k}\vert\boldsymbol{x}_{k-1}^{i},\boldsymbol{z}_{k})$   [Note: this is to integrate the multi-strain SIRS model forward 1 step, which generate a prediction]   - Assign the particle a weight, $w_{k}^{i}$, according to:   $w_{k}^{i}\propto w_{k-1}^{i}\frac{\pi\left( \boldsymbol{z}_{k} \vert\boldsymbol{x}_{k}^{i} \right)\pi\left( \boldsymbol{x}_{k}^{i} \vert\boldsymbol{x}_{k-1}^{i} \right)}{\pi\left( \boldsymbol{x}_{k} \vert\boldsymbol{x}_{k-1}^{i},\boldsymbol{z}_{k} \right)}\propto w_{k-1}^{i}\pi\left( \boldsymbol{z}_{k} \vert\boldsymbol{x}_{k}^{i} \right)$  assuming:  $\pi\left( \boldsymbol{x}_{k}^{i} \vert\boldsymbol{x}_{k-1}^{i} \right)=\pi\left( \boldsymbol{x}_{k} \vert\boldsymbol{x}_{k-1}^{i},\boldsymbol{z}_{k} \right)$  The likelihood is computed based on a multinormal distribution centered at the observations:  $\pi\left( \boldsymbol{z}_{k} \vert\boldsymbol{x}_{k}^{i} \right)\mathcal{\sim N(}\boldsymbol{\mu},\boldsymbol{\Sigma})$  [Note: this generates the updated weights/particles]  - END FOR  - Calculate total weight:  $t=sum[\left\{ w_{k}^{i} \right\}_{i=1}^{N}]$  - FOR i=1:N   - Normalize: $w_{k}^{i}=t^{-1}w_{k}^{i}$   - END FOR  (Regularization:)  - Calculate $\hat{N_{eff}} using:$  $\hat{N_{eff}}=\frac{1}{\sum_{i=1}^{N} {(w_{k}^{i})}^{2}}$  - IF $\hat{N_{eff}}<N_{T}$  [Note: in this study, N_T_=N/4]   - Resample particles according to their weights - Set new weights to 1/N   - END IF  - Perturbation: $\Theta_{m,k}\sim h_{m}(\theta\vert\Theta_{m,k-1};\delta_{m})$  - Space Reprobing:   - Sample M particle indexes to replace:   $IND={SAMPLE\{i=1:N\}}_{j}^{M}$   - Sample M values of *x*_modified_ from a proper set for the replacement:   VAL=${SAMPLE\{x_{modified}^{Lower}:x_{modified}^{upper}\}}_{j}^{M}$   - Replace the value of *x*_modified_ for particles IND=j:M   ${\{x_{modified}\}}_{j}^{M}=VAL$  [Note: here *x_modified_* is the population susceptibility]  END FOR (time steps)  END FOR (iteration) |
| --- |
