# Supplementary figures and images for: Dynamic interactions of influenza viruses in Hong Kong during 1998-2018
Source: PLoS Comput Biol. 2020 Jun 15;16(6):e1007989. doi: 10.1371/journal.pcbi.1007989 (PMC7316359; doi:10.1371/journal.pcbi.1007989)

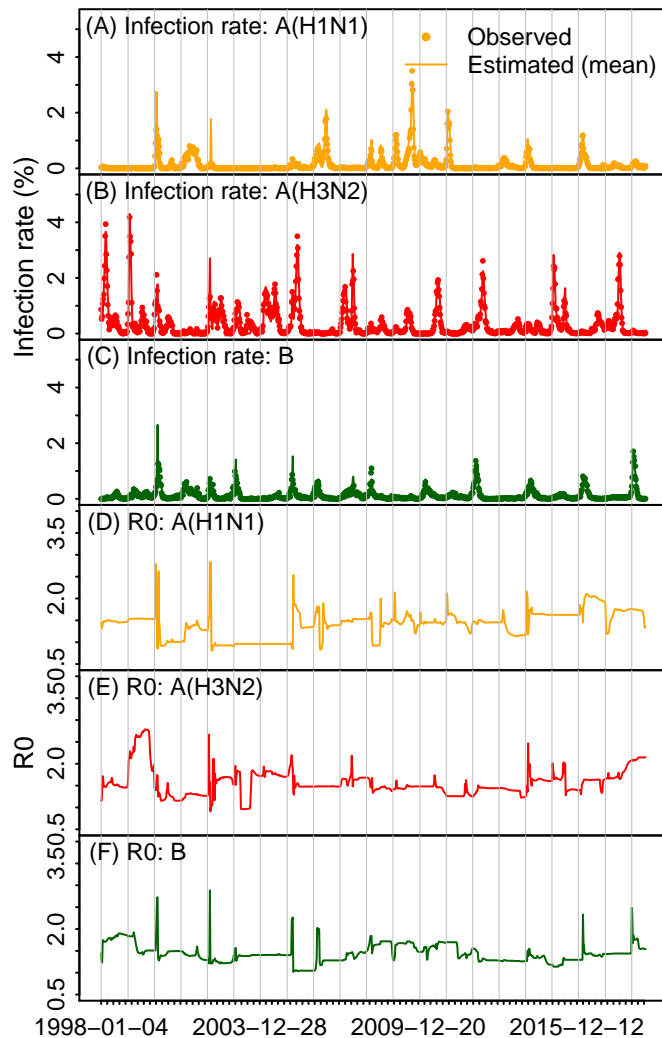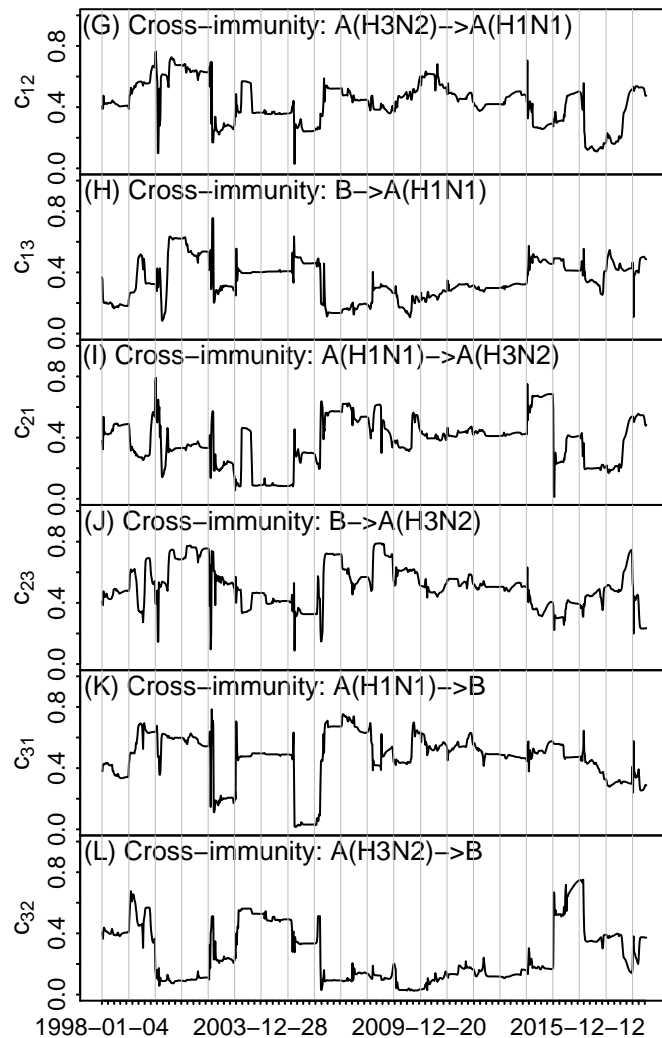

Supplement: S1 Fig — Model fits compared to the observations are shown for A(H1N1) (A), A(H3N2) (B), and B (C). Estimates of R0 are shown in D-F for the three influenza types/subtypes and strength of cross-immunity shown in G-L. (PDF) [file pcbi.1007989.s005.pdf]

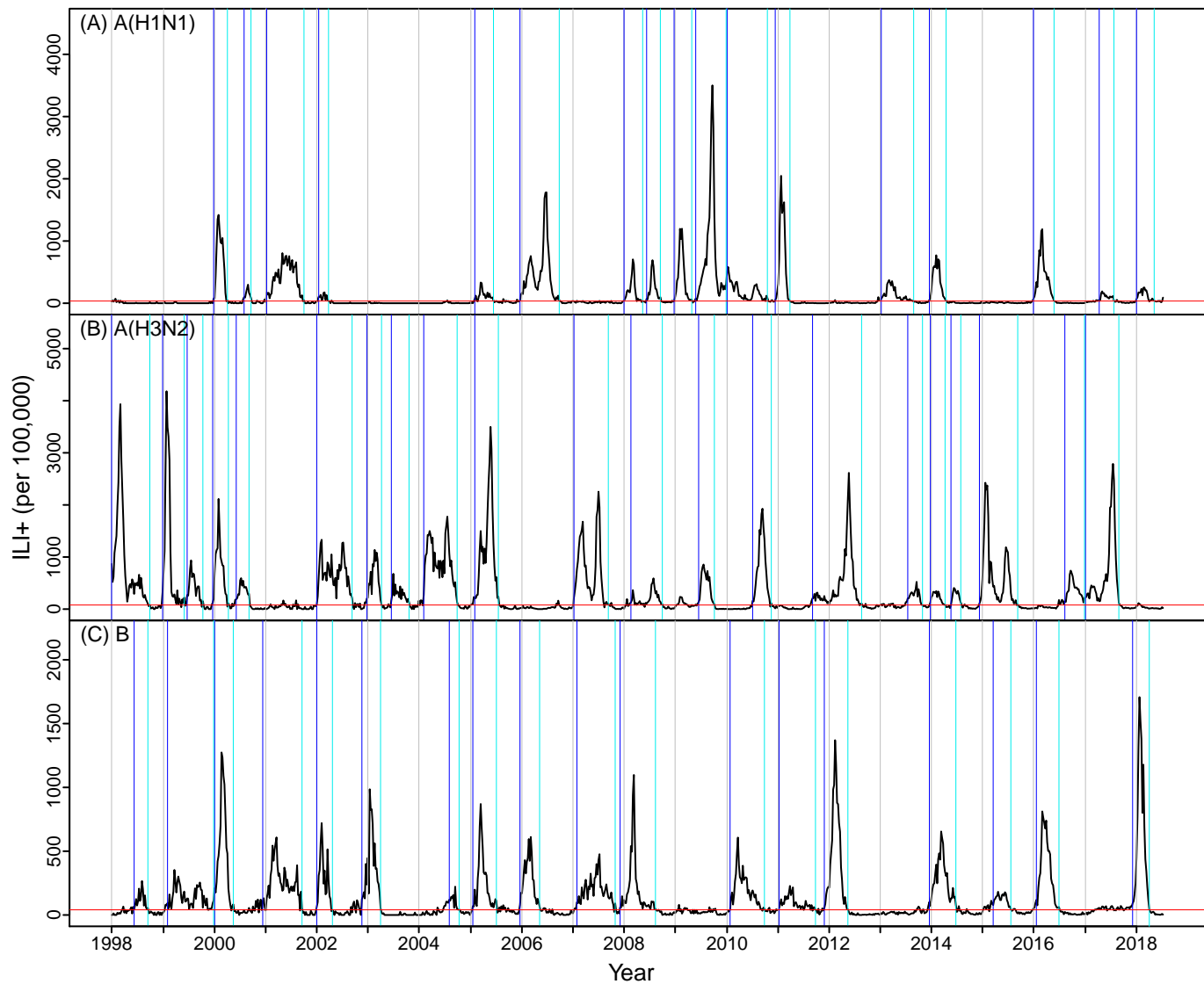

Supplement: S4 Fig — Black lines are ILI+ observations; red horizontal lines are baselines; blue vertical lines are the identified onsets; cyan vertical lines are identified endings; grey vertical lines are year divisions. (PDF) [file pcbi.1007989.s008.pdf]

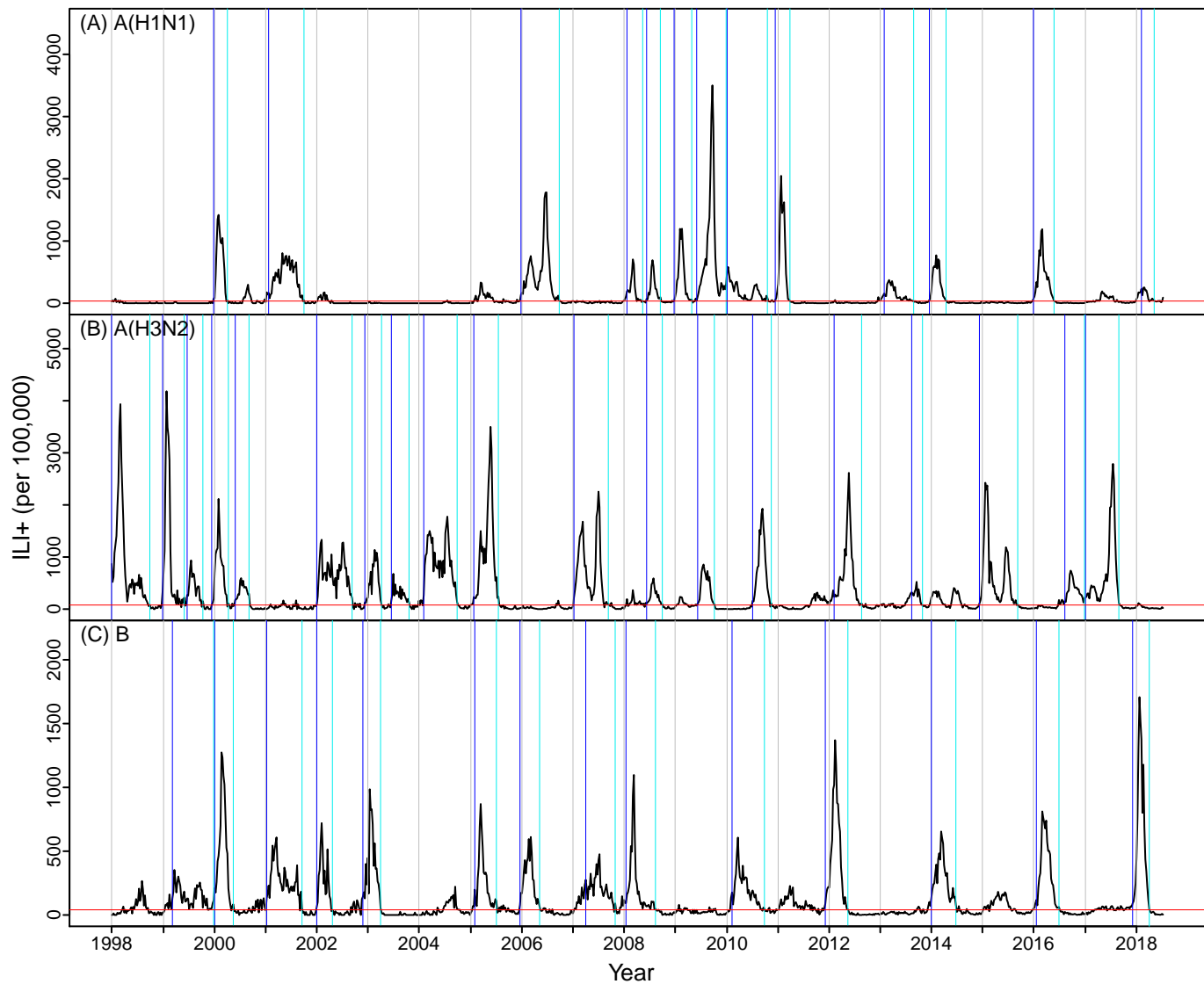

Supplement: S5 Fig — Black lines are ILI+ observations; red horizontal lines are baselines; blue vertical lines are the identified onsets; cyan vertical lines are identified endings; grey vertical lines are year divisions. (PDF) [file pcbi.1007989.s009.pdf]

**A(H1N1)**

3.9 (95% CI: -162, 103)

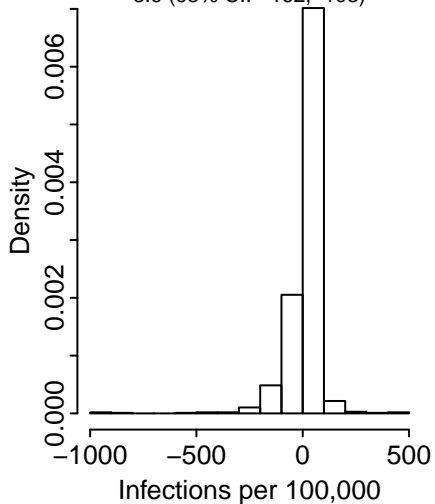**A(H3N2)**

-2.8 (95% CI: -198, 140)

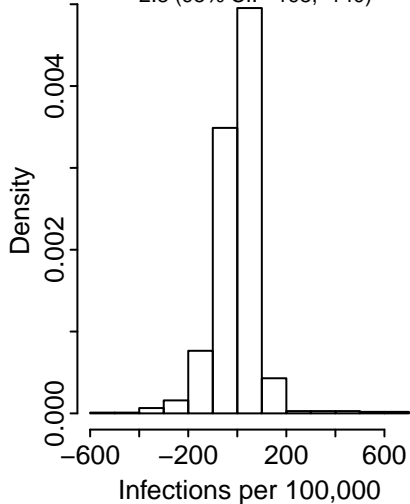**B**

9.3 (95% CI: -131, 113)

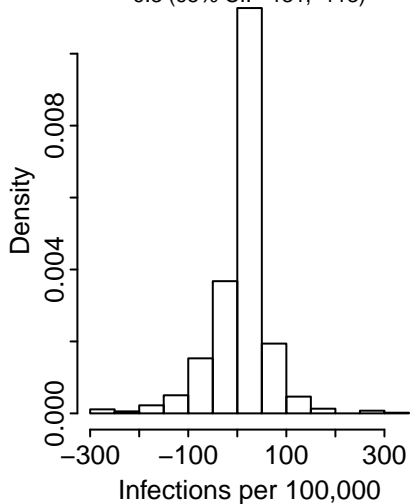

Supplement: S6 Fig — Numbers below the name of virus show the mean and 95% confidence interval in parentheses. (PDF) [file pcbi.1007989.s010.pdf]

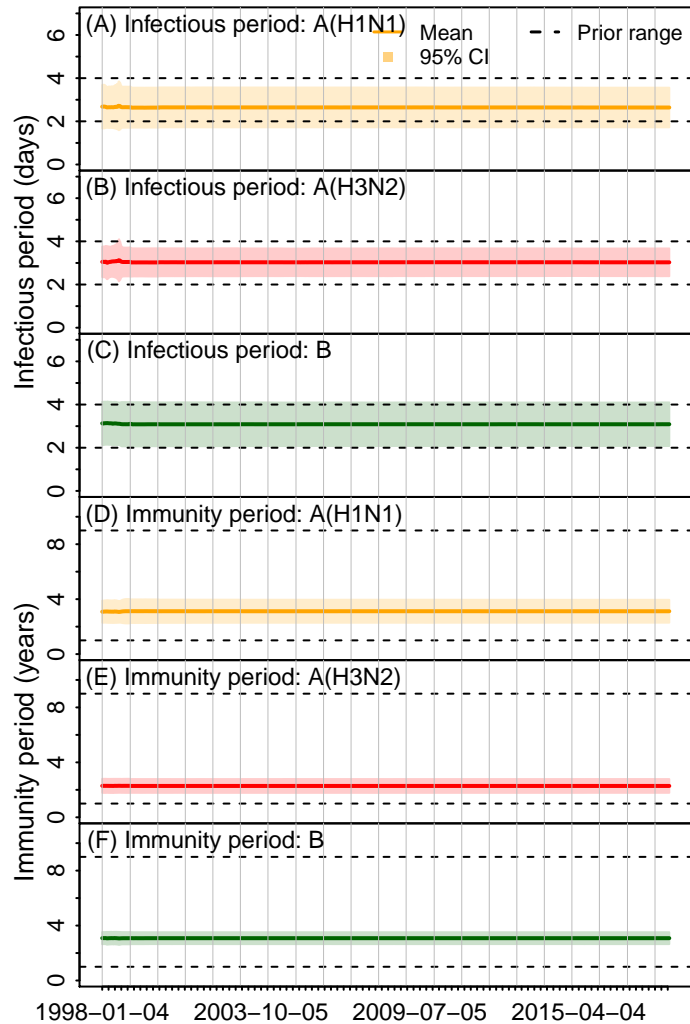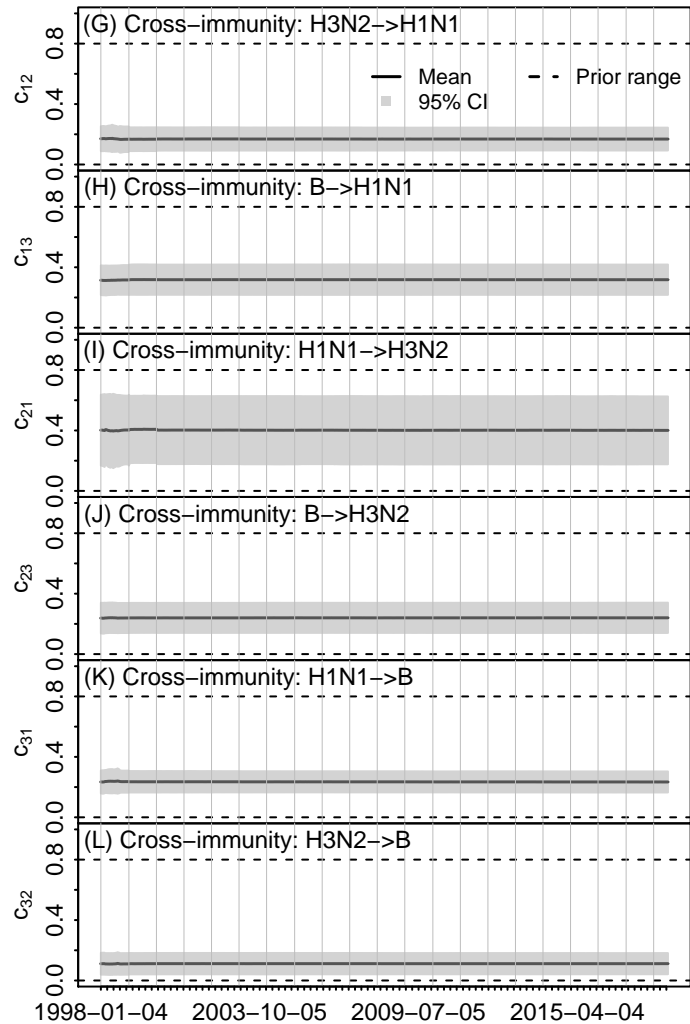

Supplement: S7 Fig — Solid lines and surrounding areas show the posterior mean and 95% CI estimates and dashed lines show the prior ranges. (PDF) [file pcbi.1007989.s011.pdf]

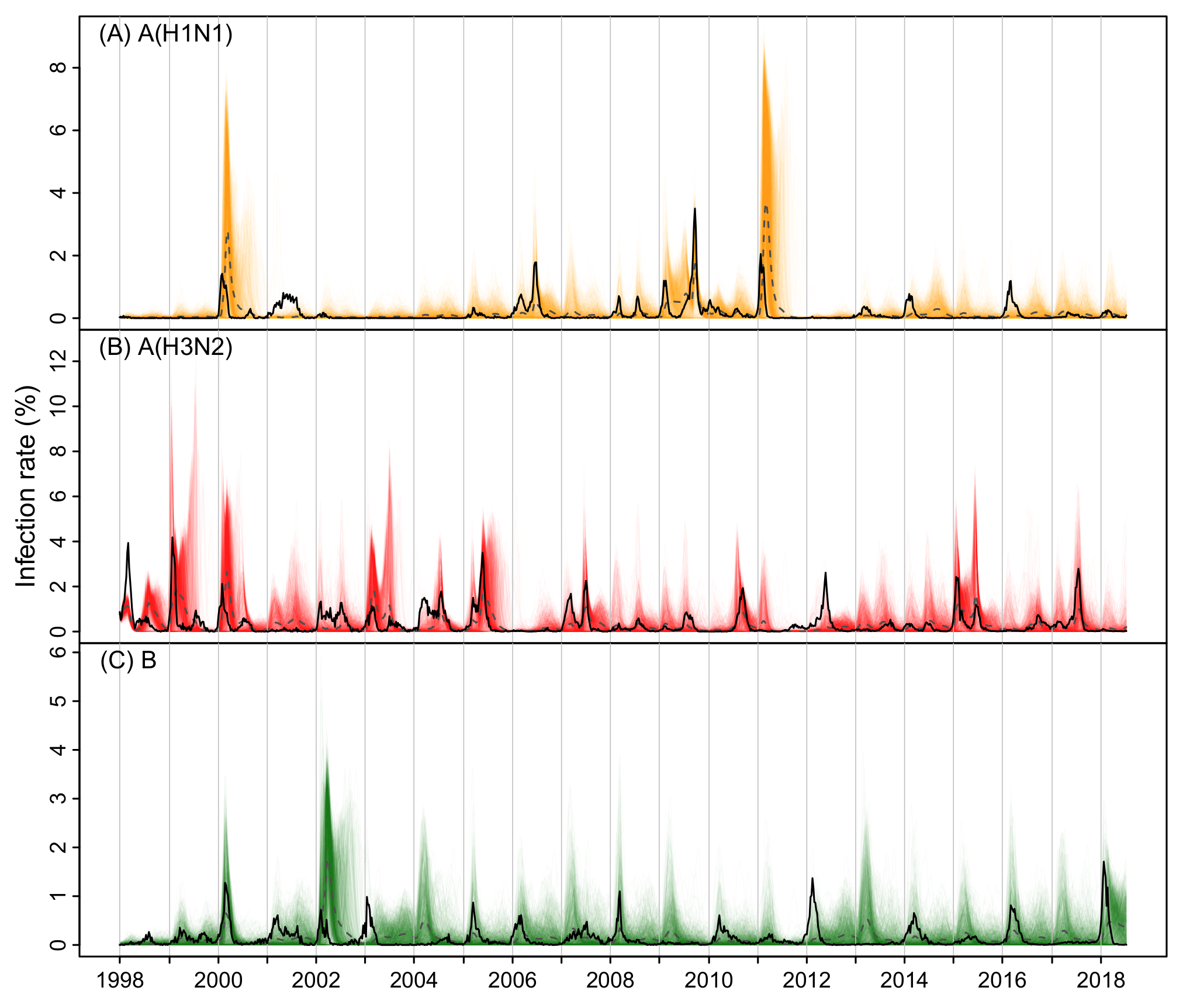

Supplement: S8 Fig — Colored lines show simulated weekly infection rates from 1000 individual stochastic model runs (A(H1N1) in orange, A(H3N2) in red, and B in green); dashed black lines show the weekly mean infection rates across 1000 simulations and solid black lines show the weekly observations for comparison. (TIF) [file pcbi.1007989.s012.tif]

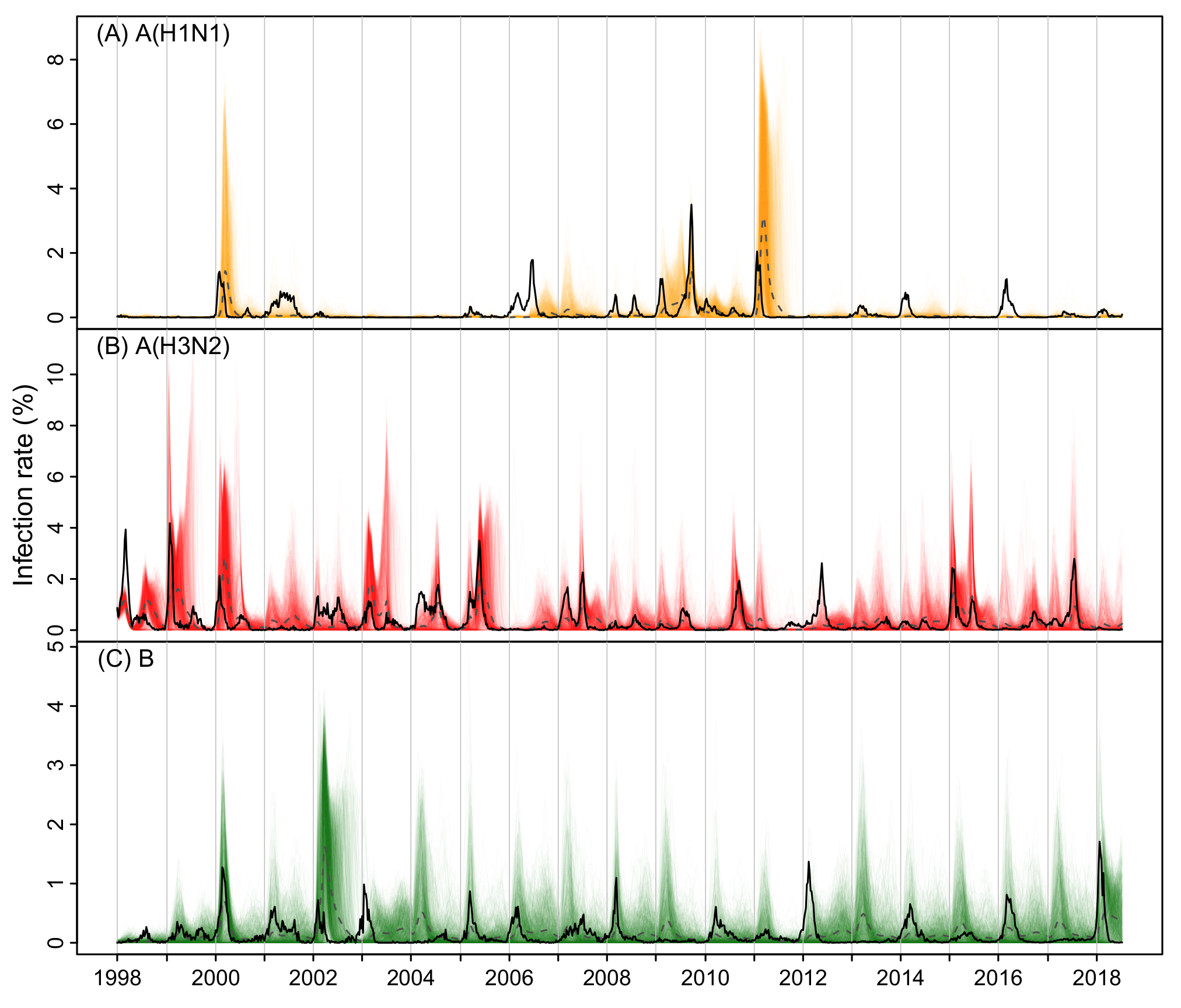

Supplement: S9 Fig — The cross-immunity parameter cH1←H3 was set to 0.5 and other parameters set to the posterior mean estimates. Colored lines show simulated weekly infection rates from 1000 individual stochastic model runs (A(H1N1) in orange, A(H3N2) in red, and B in green); dashed black lines show the weekly mean infection rates across 1000 simulations and solid black lines show the weekly observations for comparison. (TIF) [file pcbi.1007989.s013.tif]

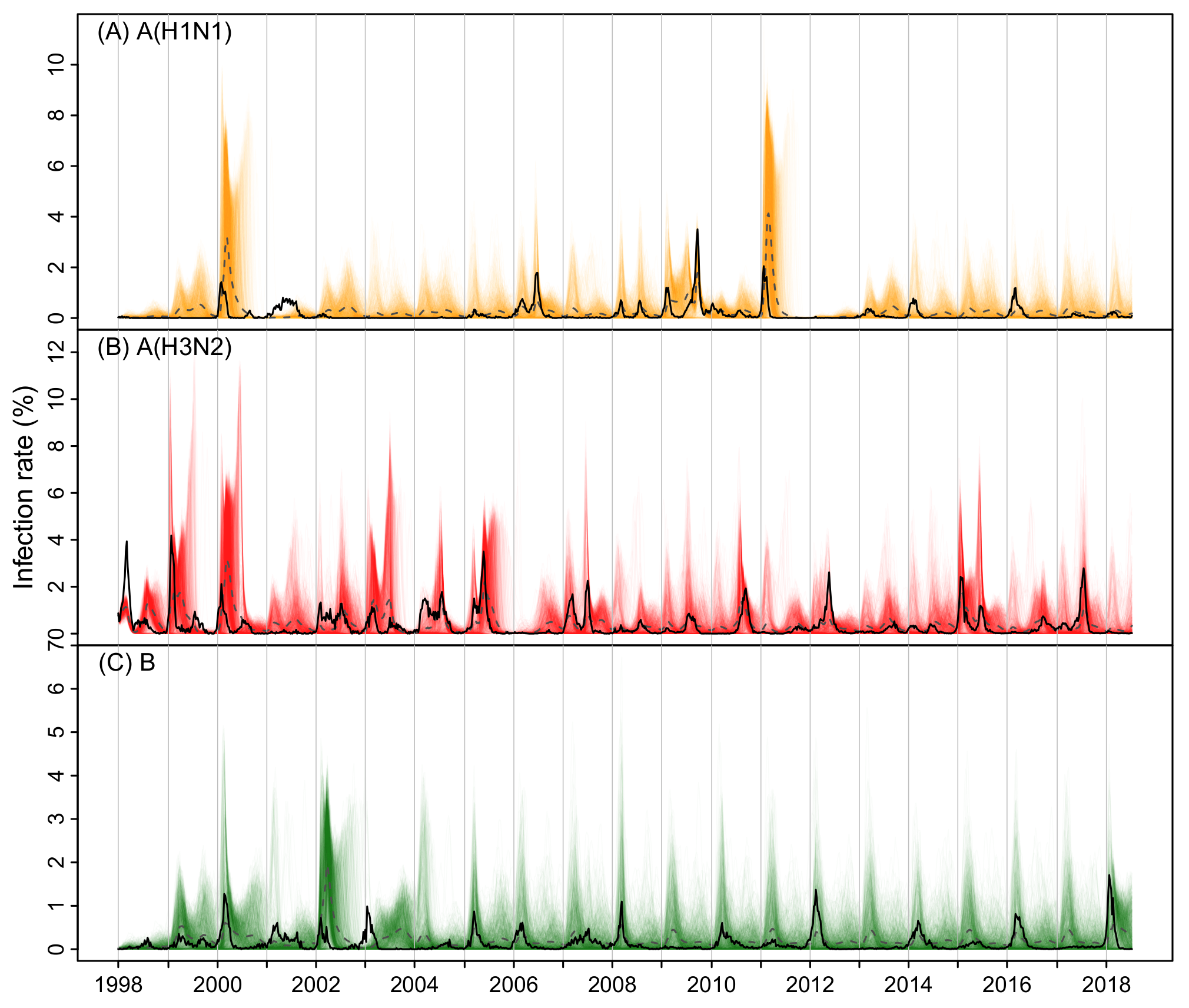

Supplement: S10 Fig — All cross-immunity terms were set to 0 and other parameters set to the posterior mean estimates. Colored lines show simulated weekly infection rates from 1000 individual stochastic model runs (A(H1N1) in orange, A(H3N2) in red, and B in green); dashed black lines show the weekly mean infection rates across 1000 simulations and solid black lines show the weekly observations for comparison. (TIF) [file pcbi.1007989.s014.tif]

(A) Number epidemics including small ones

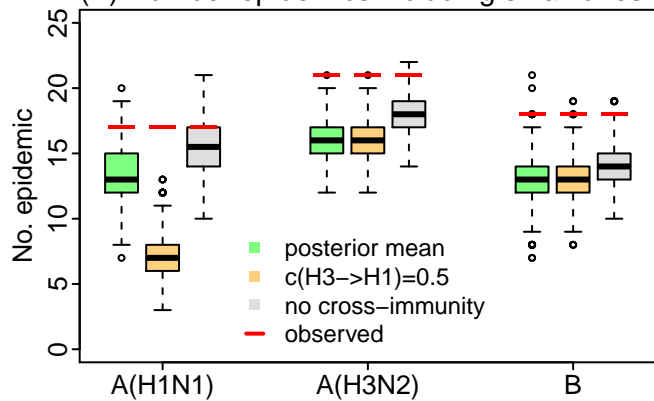

(B) Number large epidemics

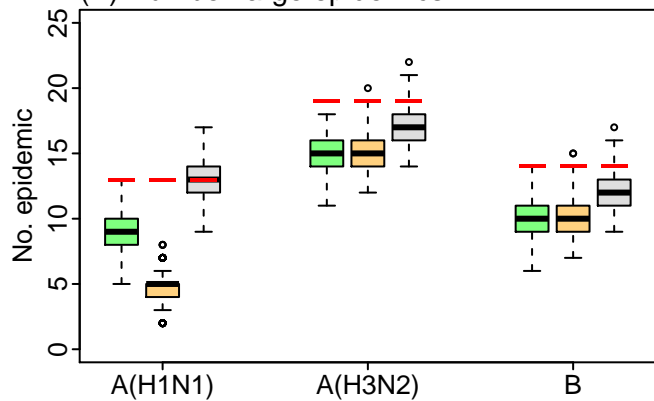

(C) Pattern of (co)circulation

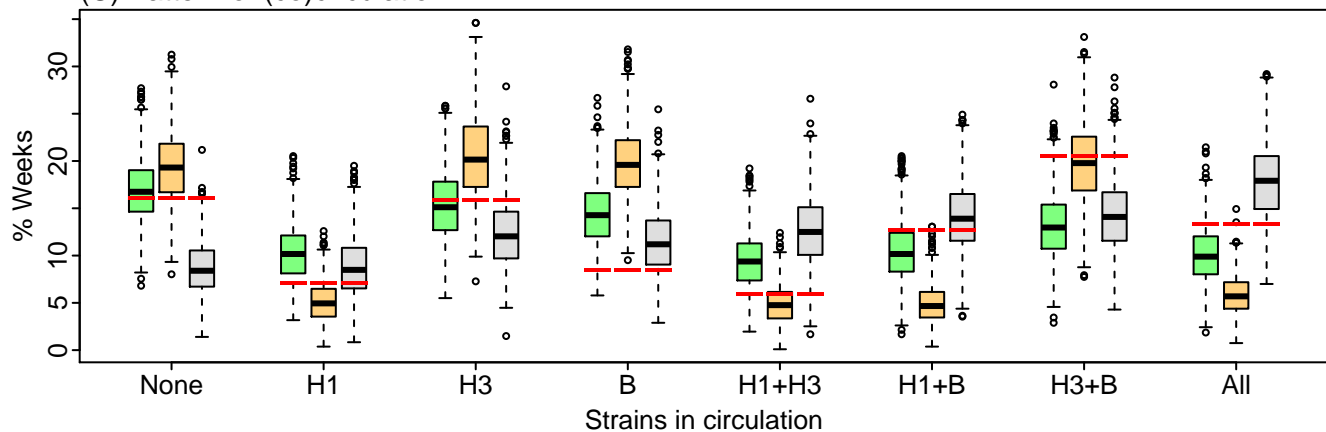

Supplement: S11 Fig — Box and whisker plots show the distributions across 1000 simulations for each cross-immunity scenario: scenario 1 as estimated in green; scenario 2 with stronger cross-immunity from A(H3N2) against A(H1N1) in orange; and scenario 3 with no cross-immunity in grey. Horizontal thick black lines show the median; box edges show the 25th and 75th percentiles; the whiskers show the full ranges and dots show outliers. Red segments show the corresponding observations in Hong Kong. (PDF) [file pcbi.1007989.s015.pdf]

Pattern of (co)circulation

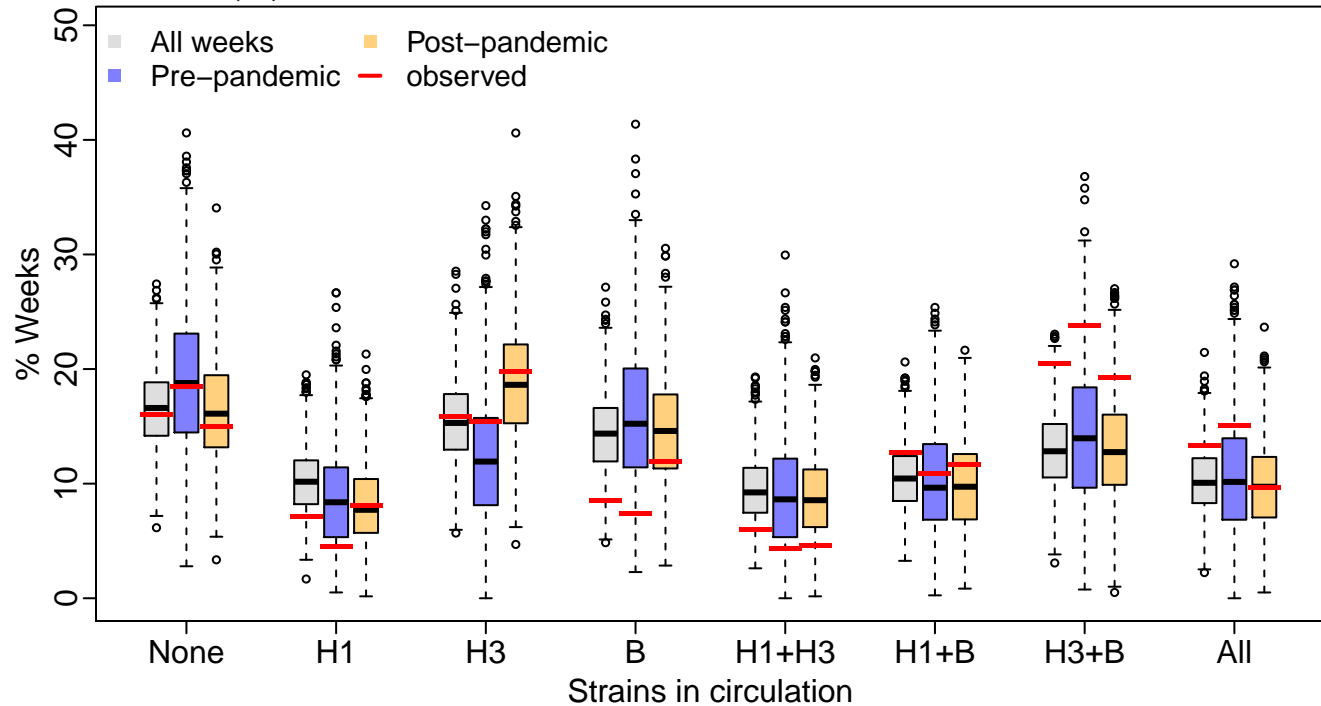

Supplement: S12 Fig — Box and whisker plots show the distributions across 1000 simulations using the parameter posterior estimates, stratified by period of time: All weeks from Jan 1998 to July 2018 in grey; Pre-pandemic from Jan 1998 to May 2009 in blue; Post-pandemic from Jan 2011 to July 2018 in orange. Horizontal thick black lines show the median; box edges show the 25th and 75th percentiles; the whiskers show the full ranges and dots show outliers. Red segments show the corresponding observations in Hong Kong. (PDF) [file pcbi.1007989.s016.pdf]

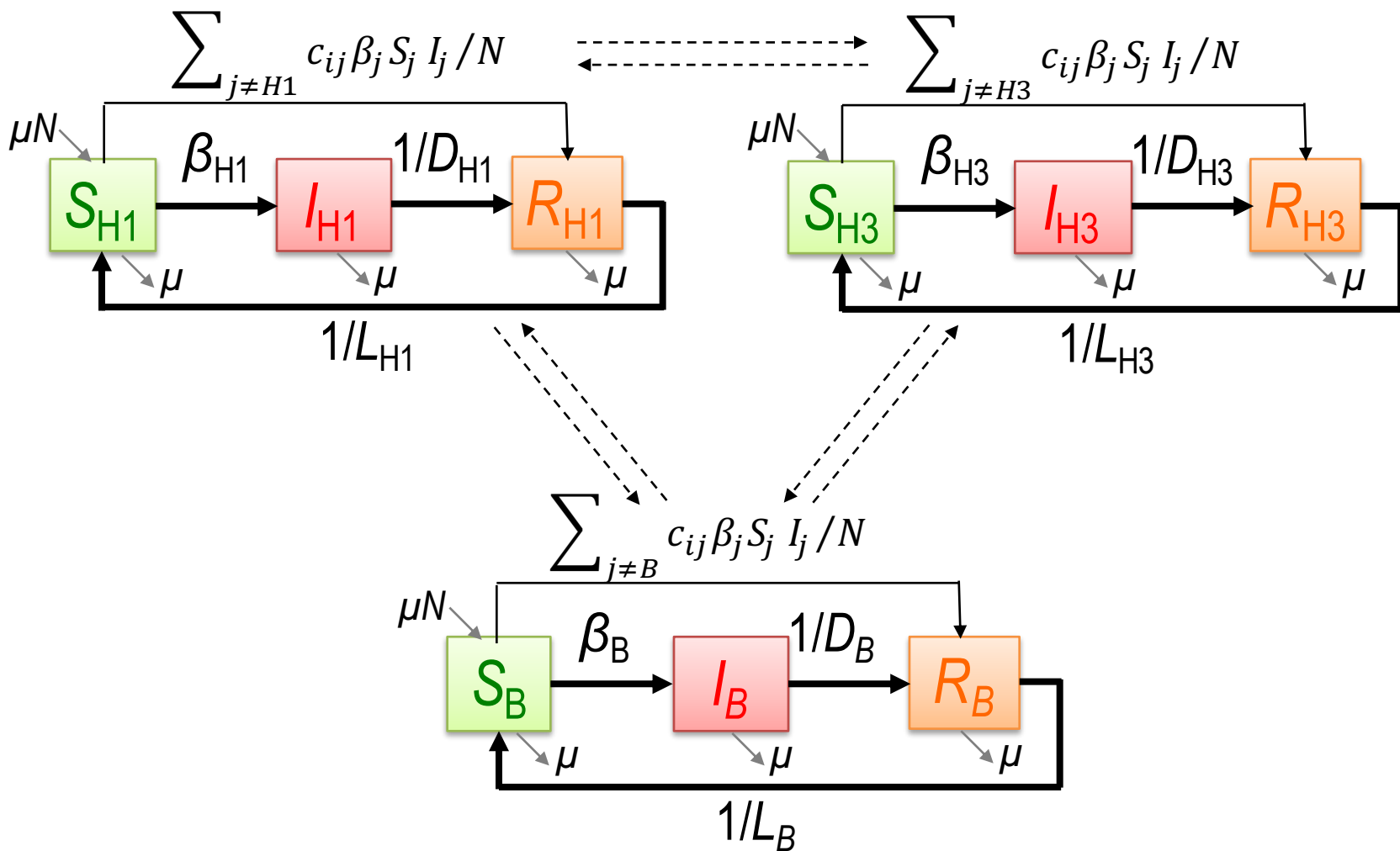

Supplement: S13 Fig — Thick solid black arrows show the epidemic dynamics for each of the three influenza viruses (H1, H3, or B): susceptible (S) individuals can get infected and move to being infectious (I), who subsequently recover (R) from the infection; due to warning immunity and influenza virus evolution, recovered individuals can lose prior immunity and return to being susceptible. While the infection processes for each virus happen independently (hence the three separate panels for the three viruses), interactions (thin dashed black arrows) among the three viruses can occur via cross-immune protection, which convert new infections by other viruses to a loss of susceptibility (thin solid black arrows). Demographic processes (birth and death) are shown by the thin solid grey arrows. See the main text for model equations and parameter notations. (PDF) [file pcbi.1007989.s017.pdf]
