# Supplementary material for: Dynamic interactions of influenza viruses in Hong Kong during 1998-2018
Source: PLoS Comput Biol. 2020 Jun 15;16(6):e1007989. doi: 10.1371/journal.pcbi.1007989 (PMC7316359; doi:10.1371/journal.pcbi.1007989)
Supplement: S3 Fig — Posterior estimates for the incidence rate and population susceptibility (A), basic reproductive number R0 (B), infectious period (C), immunity period (D), and strength of cross-immunity (E), compared to the true values. In (A) and (B), dots show the true incidence rates (in black; right y-axis), susceptibility (in blue; left y-axis), and R0 (in blue; left y-axis); the lines and surrounding areas show the mean and 95% credible interval (CI) estimates. In (A), vertical dashed black lines show the true weeks with punctuated antigenic changes and red lines show model-estimates. In (C)-(E), red stars show true parameter values; box plots show the median, 75%, and 95% CIs of posterior estimates by the model-inference system; orange dashed lines show the prior ranges tested. (PDF) [file pcbi.1007989.s007.pdf]

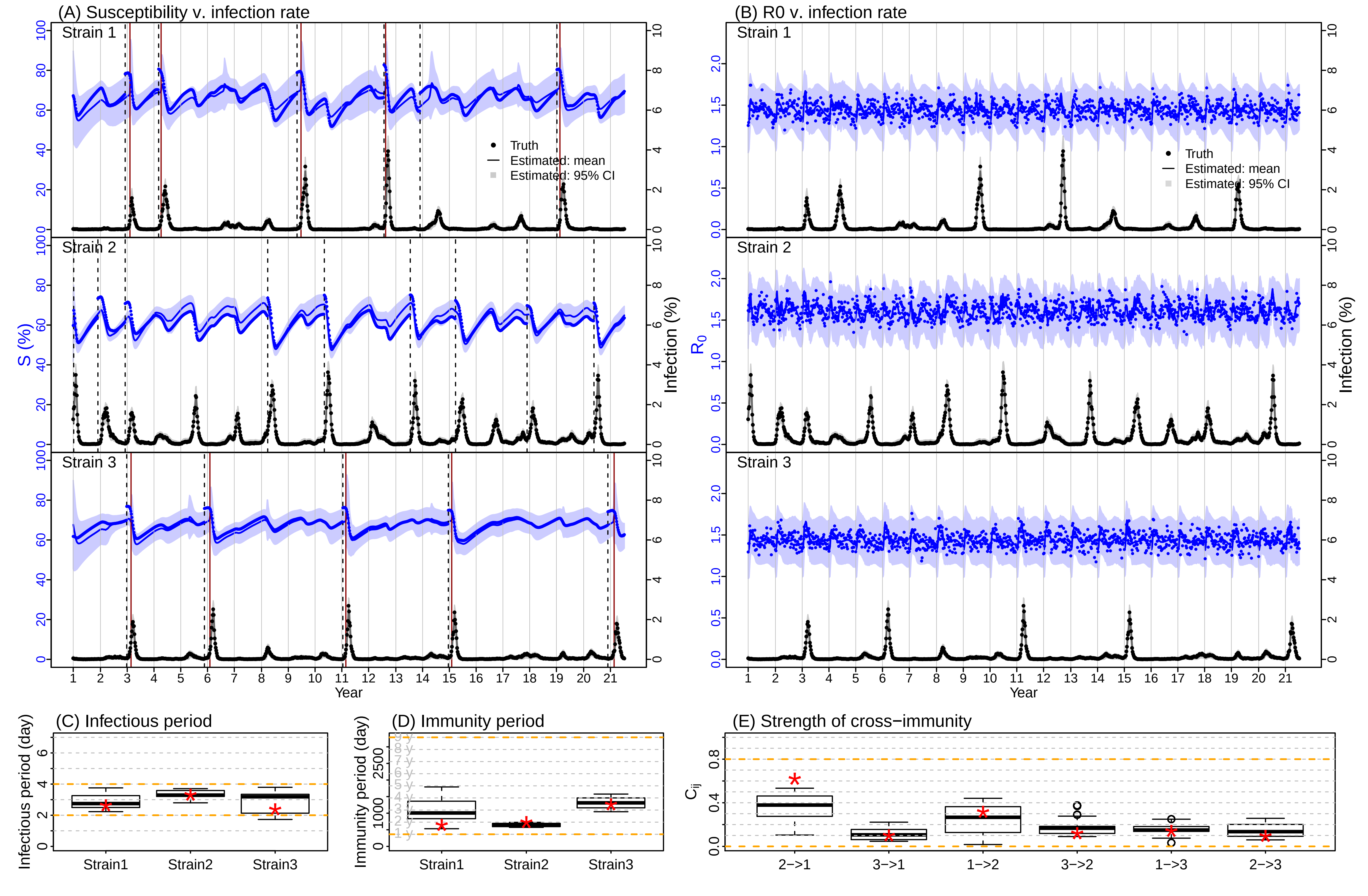

(A) Susceptibility v. infection rate
(B) R0 v. infection rate
Strain 1
Strain 2
Strain 3
Truth
Estimated: mean
Estimated: 95% CI
S (%)
Infection (%)
R0
Year
(C) Infectious period
Infectious period (day)
(D) Immunity period
Immunity period (day)
(E) Strength of cross−immunity
Cij
Strain1
Strain2
Strain3
2−>1
3−>1
1−>2
3−>2
1−>3
2−>3
